# Three-Dimensional Mid-Air Acoustic Manipulation by Ultrasonic Phased Arrays

**DOI:** 10.1371/journal.pone.0097590

**Published:** 2014-05-21

**Authors:** Yoichi Ochiai, Takayuki Hoshi, Jun Rekimoto

**Affiliations:** 1 Graduate School of Interdisciplinary Information Studies, The University of Tokyo, Tokyo, Japan; 2 Center for Fostering Young and Innovative Researchers, Nagoya Institute of Technology, Aichi, Japan; 3 Sony CSL, Tokyo, Japan; Pacific Northwest National Laboratory, United States of America

## Abstract

The essence of levitation technology is the countervailing of gravity. It is known that an ultrasound standing wave is capable of suspending small particles at its sound pressure nodes. The acoustic axis of the ultrasound beam in conventional studies was parallel to the gravitational force, and the levitated objects were manipulated along the fixed axis (i.e. one-dimensionally) by controlling the phases or frequencies of bolted Langevin-type transducers. In the present study, we considered extended acoustic manipulation whereby millimetre-sized particles were levitated and moved three-dimensionally by localised ultrasonic standing waves, which were generated by ultrasonic phased arrays. Our manipulation system has two original features. One is the direction of the ultrasound beam, which is arbitrary because the force acting toward its centre is also utilised. The other is the manipulation principle by which a localised standing wave is generated at an arbitrary position and moved three-dimensionally by opposed and ultrasonic phased arrays. We experimentally confirmed that expanded-polystyrene particles of 0.6 mm, 1 mm, and 2 mm in diameter could be manipulated by our proposed method.

## Introduction

Ultrasonic levitation method has been used to levitate lightweight particles [1], small creatures [2], and water droplets [3]. The principle of acoustic levitation was mathematically explained by Gor’kov [4] and Nyborg [5]. The potential energy *U* of an ultrasound standing wave is given by.
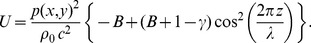
(1)


The acoustic axis coincides with the *z* axis, and *p*(*x*, *y*) is the cross-sectional sound pressure distribution. *B* is given by 3(*ρ* − *ρ*_0_)/(2*ρ*+*ρ*_0_), where *ρ* and *ρ*_0_ are the densities of a small sphere and the medium, respectively; *γ* is given by *β*/*β*_0_, where *β* and *β*_0_ are the compression ratios of the small sphere and the medium, respectively; *c* is the speed of sound in the medium; and *λ* is the wavelength of ultrasound. The force **F** acting on a sphere of volume *V* is obtained by **F** = −*V* ∇*U*. This principle has been examined using bolted Langevin-type transducers with fixed acoustic axes.

## Materials and Methods

### Phased Array

**Figure 1:**
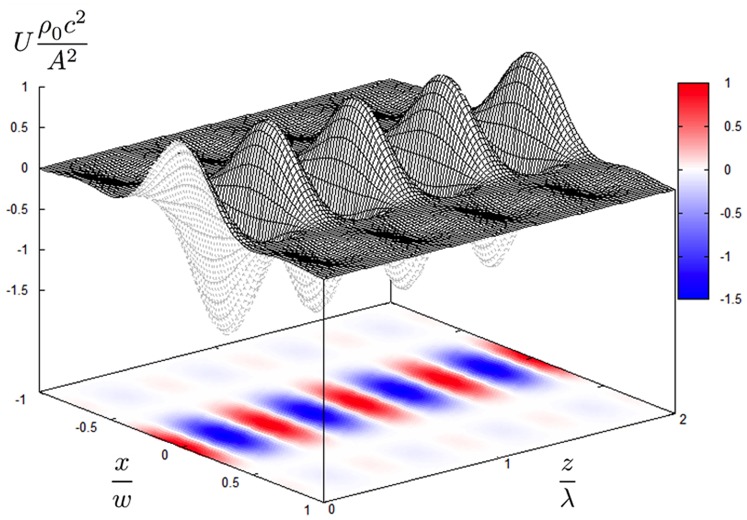
Potential energy distribution of ultrasonic standing wave. This figure is obtained based on Eqs. (1) and (2). The horizontal axes *x/w* and *z/λ* are the radial and axial directions of the beam, respectively. The vertical axis is the normalized potential energy. The gradient of this distribution gives the force on a small particle.

We innovatively employed ultrasonic phased arrays [6] as transducers. Two arrays opposed to each other were used to generate a standing wave at their common focal point. It was theoretically determined [7] that the pressure distribution of the focal point *p*(*x*, *y*) generated by a rectangular array that has *N*×*N* transducers as follows:
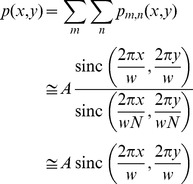
(2)where *p_m, n_*(*x*, *y*) is the sound pressure at the focal point from the (*m*, *n*)-th transducer. *A* is the RMS amplitude. The second line is the calculated result and the third line is the simplified form valid in the vicinity of the focal point. Here, the two-dimensional sinc function sinc(*x*, *y*) is defined as sin(*x*)sin(*y*)/*xy*. *w* is the diameter of the focal point given by 2*λR*/*D,* where *R* and *D* are the focal length and the side length of the rectangular array, respectively. Figure 1 shows the potential energy distribution based on Eq. (1) when *y* = 0. It is assumed here that the sphere is made of polystyrene and the medium is air. Hence, *ρ* = 1.0×10^3^ kg/m^3^, *ρ*_0_ = 1.2 kg/m^3^, *β* = 2.5×10^−10^ Pa^−1^, and *β*_0_ = 7.1×10^−6^ Pa^−1^. The figure shows that small spheres gravitate toward the acoustic axis of the ultrasound beam at its nodes.

**Figure 2:**
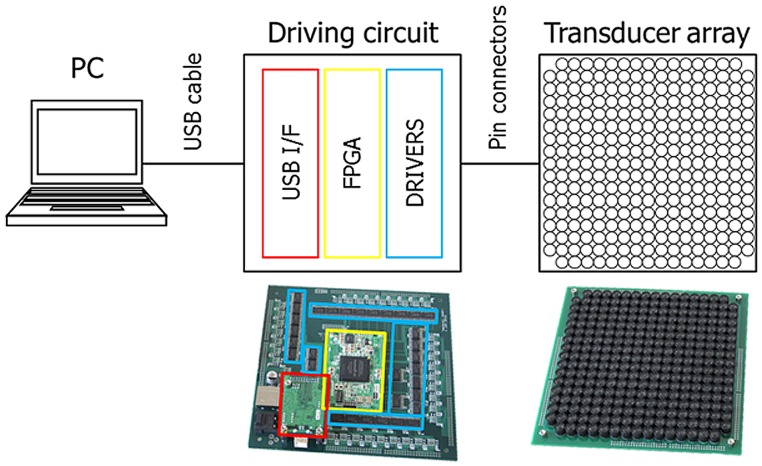
Phased array modules. (Left) The phased array modules are operated by a computer via USB. (Middle) Each driving circuit board consists of three components; USB I/F, FPGA, and Driver ICs. (Right) Each phased array module has 285 ultrasonic transducers.

The detailed specifications of the phased array (shown in Figure 2) are as follows. It consisted of 285 transducers arranged in a 170×170 mm^2^ square area and designed to generate a single focal point by adequate control of their phase differences. The resonant frequency was 40 kHz, and the sound pressure at the peak of the focal point was as high as 2600 Pa (RMS) when the focal length *R* was 200 mm. The spatial resolution of the position of the focal point was 0.5 mm, and the refresh rate was 1 kHz.

### Levitation Principle

Whymark [8] investigated the suspending force of the acoustic levitation and showed that the force *F_x_* vertical to the acoustic beam is weaker than the force *F_z_* parallel to the acoustic beam. Here, we discuss how a small polystyrene sphere can be levitated in air by the force per unit volume. We suppose that *y* = 0 and *z* = 0.25*λ* (i.e. a node). The *x* component *F_x_*/*V* is therefore obtained by determining the gradient of Eq. (1):
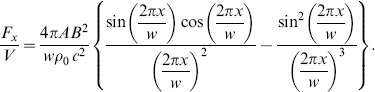
(3)


A polystyrene sphere can be levitated by this force if its density is less than *F_x_*/*Vg*, where *g* = 9.8 m/s^2^ is the gravitational acceleration. The speed of sound is 340 m/s. The amplitude *A* generated by two phased arrays has a maximum value of 5200 Pa (RMS) when the focal length *R* = 200 mm, and *w* = 20 mm. *F_x_*/*Vg* has a peak value of 5.0×10^3^ kg/m^3^ at *x* ≈ −0.2 *w*. This value is greater than the density of polystyrene, and it is therefore expected that a small sphere of polystyrene would be levitated even when the ultrasound beam is perpendicular to the gravitational force.

### Implementation

**Figure 3:**
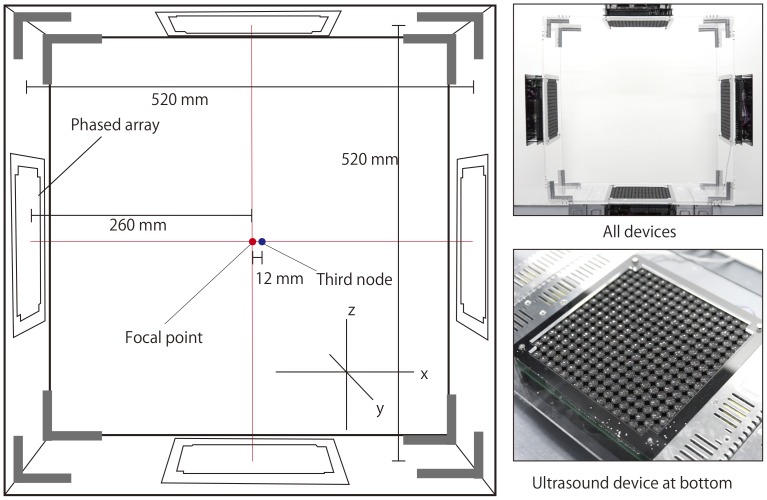
Illustration and photograph of system setup. The size of system is 520(height)×520 mm(width)×250 mm(depth). The focal point is set in the center of workspace. The labels of axis are shown in the figure: x-axis and z-axis are parallel to the device plane and y-axis is vertical to the device plane. We inserted the particles at the third node of beams that is parallel to x-axis in the stability experiments.

We developed our manipulation system with four modules of phased array, as shown in Figure 3. The surrounded area is 520×520 mm^2^. We placed the phased arrays facing each other.

We have two options of phased arrays with different frequencies (40 and 25 kHz). The position of the focal point is digitally controlled with a resolution of 1/16 of the wavelength (approximately 0.5 mm for the 40-kHz ultrasound) and can be refreshed at 1 kHz. The 40-kHz phased array consists of 285 transducers (10-mm diameter, T4010A1, Nippon Ceramic Co., Ltd.) arranged in a 170×170 mm^2^ square area. The sound pressure at the peak of the focal point is 2585 Pa RMS (measured) when the focal length R = 200 mm. The 25-kHz phased array consists of 100 transducers (16-mm diameter, T2516A1, Nippon Ceramic Co., Ltd.). The sound pressure at the peak of the focal point is 900 Pa RMS (estimated) when the focal length R = 200 mm. Using the 25-kHz phased arrays, the suspending force is much smaller while the size of the focal point is larger. In this study, we primarily use the 40-kHz phased arrays to obtain a larger suspending force.

The size and weight of a single phased array are 19×19×5 cm^3^ and 0.6 kg, respectively. It consists of two circuit boards. One is an array board of ultrasonic transducers and the other is a driving board, including an FPGA and amplifiers (shown in Figure 2). They are connected electrically to each other by pin connectors.

The phased array is controlled by a single PC via USB. The control application is developed in C++ on Windows. The PC sends the data including the coordinates of the focal point and output intensity to the driving board. The driving board receives the data, calculates adequate time delays for the individual transducers, and generates the driving signals that are sent to the transducers via the amplifiers. Modifying the time-delay calculation algorithm changes the distribution of the acoustic-potential field. The output intensity is varied using PWM control of the driving signal.

### Experimental Setup on Stability

We examined the stability of the manipulation by measuring the duration of the cyclic movement at different frequencies. The test was conducted using two types of particles, namely expanded-polystyrene spheres of diameters 0.6 mm, 1.0 mm, and 2.0 mm. In each trial, a single particle was set at the third node along one of the acoustic axes (*x* axis) from the intersection of the ultrasound beams. All the directions of movement (i.e. *x* and *y* along the acoustic axes, and *z* perpendicular to them) were tested. The focal length was set at 260 mm (Figure 2). The sound pressure was set to 70% of the maximum. The amplitude of the cyclic movement was 15 mm.

## Results

### Levitation and Manipulation

**Figure 4:**
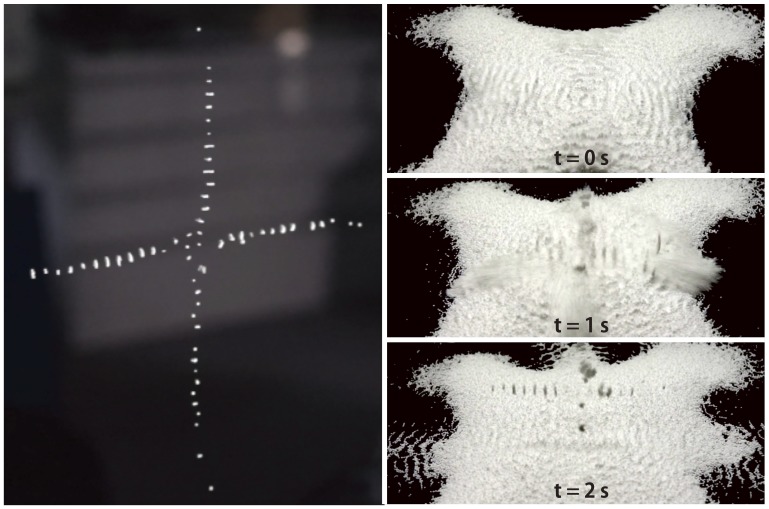
Manipulation of particles. (Left) Levitation and manipulation of particles with the vertical setup shown in Figure 3. (Right) Scooping up and holding particles with the horizontal setup in which all the ultrasonic beams are horizontally radiated.

Multiple ultrasound beams can be overlapped as shown in Figure 4 (left). The expanded-polystyrene particles are trapped at the nodes of both ultrasound beams. The interval between the trapped particles is about 4 mm, which is about half the wavelength of a 40-kHz ultrasound. It can be observed that the particles are more stably levitated using this configuration compared to using a single beam. When the beam moves through a mass of particles, the particles are scooped up and held in the beam as shown in Figure 4 (right).

### Stability

**Figure 5:**
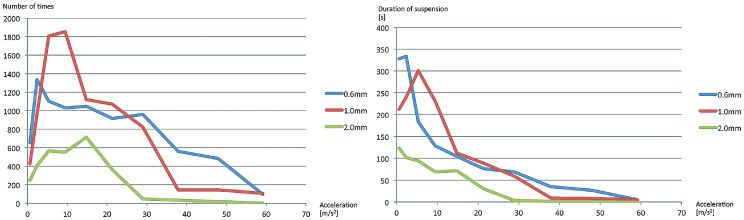
Results of stability experiments on different sizes of particles. The horizontal axis shows the maximum acceleration (cm/s^2^) within back-and-forth motion. The blue, red, and green lines show the results of 0.6 mm, 1.0 mm, and 2.0 mm, respectively. (Left) The vertical axis shows the average number of times (the duration divided by the periodic time). (Right) The vertical axis shows the average duration of suspension [s]. Both show the same experimental results.

**Figure 6:**
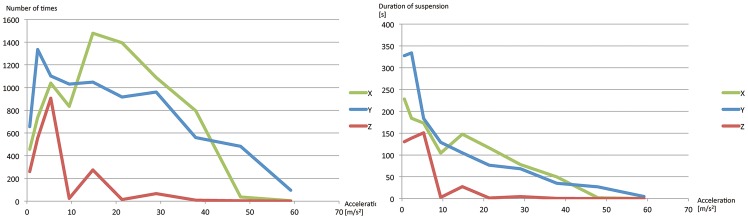
Results of stability experiments on different directions of movement. The horizontal axis shows the maximum acceleration (cm/s^2^) within back-and-forth motion. The green, blue, red lines show the results of along x-, y-, and z-axes. (Left) The vertical axis shows the average number of times (the duration divided by the periodic time). (Right) The vertical axis shows the average duration of suspension [s]. Both show the same experimental results.

The experimental results are shown in Figure 5 and 6. We moved an expanded-polystyrene particle cyclically back and forth for each trial at the maximum displacement *A* = 1.5 cm and the frequency *f* [Hz] controlled in steps of 1 Hz. The particle fell from the acoustic beam after a while. The results are shown as the average duration of suspension [s] of five trials vs. the maximum acceleration of the motion [cm/s^2^] both in Figure 5 and 6. The maximum acceleration *a* [m/s^2^] was calculated as *a* = *A*(2*πf*)^2^. Figure 5 is for different sizes of a particle (0.6 mm, 1.0 mm, and 2.0 mm) and Figure 6 is for different directions of the movement (x, y, and z axes) of a 0.6-mm-diameter particle.

Figure 6 shows that manipulation along *y* axis was more stable than the others. We speculate that the manipulations along *x* and *z* axes strongly suffer from the discontinuity of the driving signals in changing the focal length. Figure 5 shows that the 0.6-mm-diameter particles are more stable than the 2.0-mm-diameter particles at higher accelerations. This suggests that larger particles tend to fall from the nodes of the standing wave. One possible explanation is as follows. The shape of the potential field within the nodes of the standing wave affects the stability of movements. In a cone-shaped potential field, a particle suffers from a centripetal force which elicits the vibration. This vibration decreases the stability.

Next, the work space was studied. In the case of movement along one of the acoustic axes, the manipulated particles could approach the ultrasound array to within 60 mm, but fell when they approached nearer. In the case of the movement perpendicular to the acoustic axes, the particles at the more distant nodes fell earlier when they moved away from the centre of the system. A particle at the intersection of the ultrasound beams fell when it came to within 330 mm of the centre.

## Discussion

There are some factors to be considered in choosing the manipulation target, namely the size and material. The size of the manipulation target is determined by the distribution of the potential energy, and a light material is required. The internal force is also an important factor in selecting the material; for example the electrostatic force determines the maximum number of particles that can be at a single node, and the surface tension of the fluid determines the size of droplets that can be levitated.

This method is applicable to the industrial fabricating for the manipulation purpose, graphical application for floating screen, medical application for evaporation, and so on.

## Conclusion

In conclusion, we have demonstrated an extended acoustic manipulation by which millimetre-sized particles can be levitated and moved three-dimensionally by localised ultrasonic standing waves generated by ultrasonic phased arrays. In addition to the presented examples, we also tested other small objects such as a feather and droplets of alcohol and a colloidal solution.

In a future work, we will use 25 kHz transducers instead of the 40 kHz type. Then the 4-mm node intervals by 40 kHz transducers will be extended to 8 mm. This would enable the manipulation of larger particles.

It has not escaped our notice that our developed method for levitation under gravity suggests the possibility of developing a technology for handling objects under microgravity.

## Supporting Information

Video S1 Video Documentation. The video shows the particle manipulation, system overview, visualization of standing waves, and procedure of experiments. The length of this video is 2 minutes and 11 seconds.
(MOV)
