# Correction: Three-Dimensional Mid-Air Acoustic Manipulation by Ultrasonic Phased Arrays

**DOI:** 10.1371/journal.pone.0102525

**Published:** 2014-07-09

**Authors:** The PLOS ONE Staff

Attribution for the music used in Video S1 is missing. The music is republished under a CC BY license from the Classical Music Sound Library, http://classical-sound.seesaa.net/, created by Ando Towa.
